# Oil Biodegradation and Bioremediation in Cold Marine Environment

**DOI:** 10.3390/microorganisms11051120

**Published:** 2023-04-25

**Authors:** Jaak Truu

**Affiliations:** Institute of Molecular and Cell Biology, Faculty of Science and Technology, University of Tartu, Riia 23, 51010 Tartu, Estonia; jaak.truu@ut.ee

Petroleum hydrocarbons pose a substantial threat to marine ecosystems. Their release into the ocean due to human activities, such as drilling, manufacturing, storage, and the transportation of crude oil and oil products, raises significant concerns. As climate change opens up new shipping routes in the Arctic, the risks of oil spills resulting from oil and gas exploration, as well as increased marine traffic in the region, have grown considerably [1].

One challenge in studying the biodegradation of oil in cold marine environments is identifying and characterizing the oil-degrading microbial species and their potential interactions. Different oil components possess diverse chemical structures and properties, necessitating various microbial species with specific enzymes and pathways for degradation. Furthermore, the microbial consortia degrading oil in seawater and sediments are both dynamic and complex, with their composition and activity subject to fluctuations depending on environmental conditions and the availability of nutrients and oxygen. While it is confirmed that oil biodegradation can occur at low and sub-zero temperatures, knowledge about the extent of this process and the specific microorganisms involved in cold marine environments remain limited [2]. As a result, there is an urgent need for further research on the capabilities of individual microorganisms and microbial communities to degrade oil compounds in cold marine settings.

One approach to mitigating the negative impact of oil pollution in the marine environment is bioremediation, which involves using microorganisms to degrade or detoxify pollutants. Bioremediation can be used to clean up oil spills and reduce the long-term effects of petroleum hydrocarbons on the marine ecosystem. However, to develop effective bioremediation strategies, it is crucial to understand the microbial communities involved in the process and their response to different environmental conditions [3].

The objective of this Special Issue was to gather articles investigating microbial taxa and consortia with specific metabolic pathways involved in the biodegradation of oil compounds within various marine compartments in cold marine environments. The focus was on the abilities of marine microbes at taxonomic, functional, and genomic levels to react to and degrade hydrocarbons originating from oil spills at low and sub-zero temperatures. Furthermore, the Special Issue explored the development and application of oil bioremediation techniques to address marine oil spills in cold seawater and ice-affected areas.

The research article by Peeb et al. [4] assessed the structure and diversity of the microbial community and estimated its genetic potential for oil hydrocarbon degradation in Arctic seawater, as well as in uncontaminated and crude oil-encapsulating sea ice. In their article, Vigneron et al. [5] investigated the microbial community present in the aqueous phase of a subsea oil-storage structure located in the North Sea's Brent oil field, which is currently in the process of decommissioning. The microbial community was found to be dominated by organisms related to Dethiosulfatibacter and Cloacimonadetes, with a strong potential for degrading low-molecular-weight aromatic hydrocarbons.

Two papers addressed oil bioremediation in the cold marine environment. The effect of biostimulation on microbial community abundance, structure, dynamics, and the metabolic potential for oil hydrocarbon degradation in oil-contaminated Arctic seawater were studied by Nõlvak et al. [6]. The second paper, by Tomasino and co-authors [7], addressed petroleum pollution in cold, deep marine sediments. Using the deep-sea sediment samples from the North Atlantic Ocean, they identified bacterial strains with the potential for the enhanced bioremediation of petroleum hydrocarbon-polluted sediments.

The study by Zakaria et al. [8] investigated a psychrotolerant marine bacterial consortium from the northwest Antarctic Peninsula, optimizing its growth conditions to enhance diesel hydrocarbon degradation. The research demonstrated a significant mass reduction of diesel within a 6-day incubation period, providing further evidence for the existence of native hydrocarbon-degrading bacteria in uncontaminated Antarctic seawater.

Another study from Antarctica characterized the phenanthrene-degrading bacterial strain of *Dietzia psychralcaliphila* J1ID isolated from sediments near Deception Island [9]. Based on whole genome sequencing results, this strain has a wide range of catabolic genes involved in biodegradation for toxic aromatic compounds.

In summary, this Special Issue features a series of research papers showcasing how recent advancements in molecular and data analysis techniques, coupled with cultivation-based methodologies, have significantly enhanced our understanding of marine microbial communities and their relationship with oil exposure and biodegradation in cold marine environments. As microbial communities play a critical role in the biodegradation of oil compounds in cold marine environments, the information provided by these studies allows for better predictions of oil biodegradation capacities in seawater, sediments, and coastal areas. Additionally, the findings from these studies contribute to the development of targeted mitigation strategies to the minimize negative impacts on vital ecosystem functions, such as nutrient cycling and hypoxia, in cold marine ecosystems. As our knowledge of marine microbial communities and their relationship with oil exposure and biodegradation expands, future research should focus on the microbial ecology of oil biodegradation in the presence of co-contaminants, especially microplastics. This would involve understanding the synergistic or antagonistic effects of these co-contaminants on biodegradation processes. Moreover, assessing the impact of marine oil pollution on animal microbiomes will provide insights into the potential consequences of oil pollution for marine life and ecosystem health.

Furthermore, there is a need to develop and optimize bioremediation approaches for responding to marine oil spills in cold climates and ice-affected regions. This could involve the identification and use of native psychrophilic or psychrotolerant microorganisms, as well as the implementation of novel strategies to improve the efficiency of bioremediation efforts. By addressing these research gaps, we can better protect fragile, cold marine environments and maintain the health of these vital ecosystems in the future.
